# Algorithmic procedure for retrieving calorific contents of marine phytoplankton from space

**DOI:** 10.1016/j.mex.2021.101579

**Published:** 2021-11-16

**Authors:** Shovonlal Roy

**Affiliations:** Department of Geography and Environmental Science, University of Reading, Whiteknights, Reading RG6 6AB, U.K

**Keywords:** Ocean colour, Satellite algorithm, Phytoplankton carbohydrate, Phytoplankton protein, Phytoplankton lipid, Phytoplankton energy content

## Abstract

Microscopic marine phytoplankton are crucial for the survival of marine animals and sustainability of marine food webs. Developing our capability to estimate and monitor the calorific value of marine phytoplankton in the global ocean is, therefore, invaluable. Using satellite remote-sensing, Roy (2018) reported the first global estimates of phytoplankton macromolecular concentrations based on a novel semi-analytical ocean-colour algorithm. The complete retrieval method builds on semi-analytical computational steps that were developed independently and were customised for ad-hoc applications to certain ocean-colour repository. Given the increasing interest in applying this method in local, regional and global scales, the technical details and customizations associated with the method is presented in this paper.

• The method is presented with extensive level of technical details with illustrations, so that the users can follow this standalone document and implement the method on a coding platform of their choice.
• The method can be implemented on any satellite ocean-colour repository, and at any spatial or temporal resolution.
• Given that a wide variety of software packages are used in the field of ocean-colour algorithms and that the users may be constrained with certain coding platforms, no specific software package is made mandatory to implement the method.

## Graphical abstract

**Figure:**
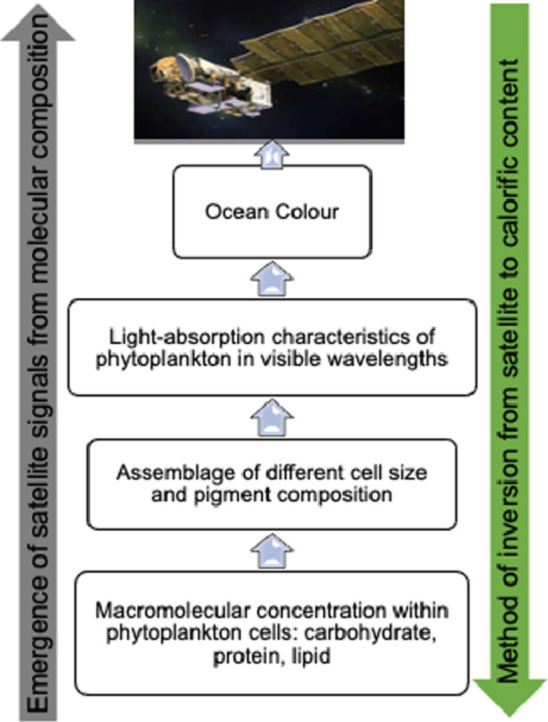

Specifications Table

**Table:** 

Subject Area:	Environmental Science
More specific subject area:	Ocean Sciences
Method name:	Semi-analytical ocean-colour algorithm for phytoplankton calorific contents
Name and reference of original method:	Roy, S. (2018) Distributions of phytoplankton carbohydrate, protein and lipid in the world oceans from satellite ocean colour. *The ISME Journal*, 12 (6), 1457-1472
	Roy, S., Sathyendranath, S., Bourman, H. and Platt, T. (2013) The global distribution of phytoplankton size spectrum and size classes from their light-absorption spectra derived from satellite data. *Remote Sensing of Environment*, 139, 185-197
	Roy, S., Sathyendranath, S. and Platt, T. (2011) Retrieval of phytoplankton size from bio-optical measurements: theory and applications. *Journal of Royal Society Interface*, 8 (58), 650-660
Resource availability:	MATLAB script as Supplementary Materials

## Method details

Calorific value of microscopic phytoplankton in the ocean is determined by their cellular macromolecular composition. The elemental composition of carbon, nitrogen and phosphorous in phytoplankton cells i.e., the stoichiometric ratio, varies across oceanographic regions [4], [9], and this variation alters the nutritional quality of phytoplankton as food to the marine grazers [5], [16]. To estimate and monitor the calorific contents of marine phytoplankton on a global scale, satellite remote-sensing is invaluable. A comprehensive method to derive calorific contents from ocean colour, captured by the Earth-Observation satellites, is described here. This method was originally introduced and applied in a study by Roy [11]. Some essential components of this bio-optical method was independently developed in previous studies by Roy el al.  [12], [13], and some of the steps were customised for ad-hoc applications to state-of-the-art satellite observations of ocean. The method is generic, in the sense that it is applicable to any satellite ocean-colour database, for retrieving the concentrations of phytoplankton carbohydrate, protein and lipid, and hence the total energy value of phytoplankton, on local, regional or global scales.

### *Method overview*

**Fig. 1:**
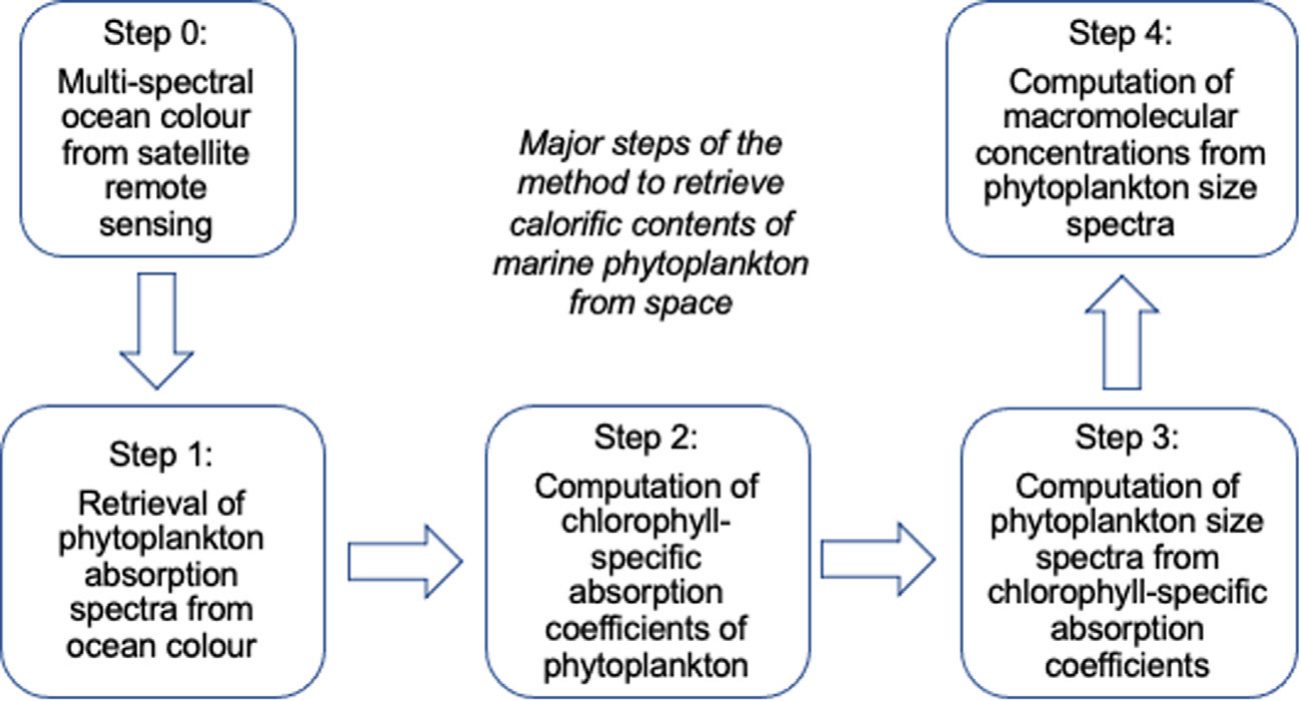
Flow diagram showing the major steps of the method for retrieving calorific contents of marine phytoplankton from satellite remote-sensing of ocean colour. The steps are described in details in the text.

The method relies on the optical fingerprints of the living phytoplankton cells in the ocean, which is captured implicitly in ocean colour by multi-spectral satellite sensors. In a nutshell, from the raw ocean-colour data, calorific contents of phytoplankton (concentrations of carbohydrate, protein, lipid) can be retrieved following four major steps as shown in Fig. 1. Multi-spectral ocean-colour data are generally freely available from satellite repositories managed by e.g. NASA and European Space Agency. In the first step, the light-absorption spectra of living phytoplankton in the visible light (i.e., wavelength 400-700 nm) is retrieved from remote-sensing reflectances using the so-called Inherent Optical Properties (IOP) algorithms. In the second step, the absorption coefficient of the main light-absorbing pigment inside phytoplankton cells i.e., chlorophyll-a (Chl-a) is computed from the phytoplankton absorption spectra. Following this, in the third step, information on cell-size distribution within a phytoplankton community is computed from the absorption coefficients of chlorophyll. Finally, in the fourth step, the macromolecular concentrations i.e. the concentrations of carbohydrate, protein and lipid are computed from the phytoplankton size spectra. The method further allows partitioning of macromolecular concentrations into generic, user-defined, phytoplankton size classes (PSCs).

### *Technical details*

In the following, the major steps of the method are described with technical details. All notations used in the method are described at first use, and for the benefit of the users, those are also compiled and described on Table 1.

**Table 1:** Notations used in the method and their meanings.

Notation	Meaning
D	Diameter of a phytoplankton cell
λ	Wavelength of incident light
$a_{ci}$	Absorption coefficient of the cell material made up of chlorophyll-a pigment
$a_{ci}^{*}$	Specific-absorption coefficient of Chl-a inside a cell m${}^{2}$
$c_{i}$	Concentration of Chl-a per unit volume of a phytoplankton cell
$c_{0}$	Proportionality constant in size-scaled intra-cellular concentration
m	Size-scaling exponent of intra-cellular chlorophyll concentration
$\rho_{c}$	Optical thickness of intact phytoplankton cell
$Q_{a}$	Absorption efficiency of a phytoplankton cell
$B_{total}$	Concentration of chlorophyll-a in a phytoplankton sample
$a_{ph}$	Total absorption coefficient of phytoplankton
$a_{chl}$	Total absorption coefficient of Chl-a only
$a_{u}$	Total absorption coefficient of pigments other than Chl-a
$a_{ph}^{*}$	Absorption coefficient of phytoplankton normalized to Chl-a
$a_{chl}^{*}$	In vivo specific absorption coefficient of Chl-a
$a^{m}$	Maximum value of absorption coefficient normalized to Chl-a
k	Constant of Junge-type power-law distribution
ξ	Exponent of the phytoplankton size spectrum
[M]	Concentration of a phytoplankton macromolecule
$\chi_{M}$	Ratio of phytoplankton macromolecule to chlorophyll concentration
$a_{M}$	Allometric constant parameter specific to the macromolecule M
$b_{M}$	Allometric exponent parameter specific to the macromolecule M
$V_{cell}$	Volume of a phytoplankton cell
$F_{M,ij}$	Fractional contribution of macromolecule M to the size class [i,j]

### *Step 1: Method to retrieve total phytoplankton absorption from satellite ocean colour*

The ocean-observing satellite sensors capture radiances that originate from the ocean surface and pass through the atmosphere. On the ocean surface, light is reflected, absorbed and scattered by the constituents of the ocean water. The inherent optical properties (IOPs) of the dissolved and suspended constituents in the ocean water determine the processes of scattering and absorption, thereby the water-leaving radiance at different wavelengths. Phytoplankton absorption coefficient is an IOP, obtaining which, from the water-leaving radiances, is not straightforward. To derive these coefficients from remote-sensing reflectance, different IOP algorithms have been developed (details of these algorithms are documented in [6]). A suitable IOP algorithm needs to be implemented first to obtain phytoplankton absorption coefficients, which are then used in Step 2. To illustrate (as in [12]), one can apply a semi-analytical inversion method developed by Carder et al. [1] (further described in [6], Chapter 9). In this method the concentration of chlorophyll-a and IOPs, including phytoplankton absorption coefficients, sum of absorption coefficients of non-algal particles plus yellow substances, and backscattering coefficient of particles at any wavelength can be computed from remote-sensing reflectance spectrum (described in [6]). The algorithm is semi-analytical, i.e, a combination of algebraic relationships and empirical relationships. This algorithm can capture the large global variability observed in specific absorption coefficients of phytoplankton, and it can be used to invert the absorption coefficients of phytoplankton at the red peak [6]. The algorithm utilises remote-sensing reflectance at four wavebands (412 nm, 443 nm, 488 nm and 547 nm), and computes specific absorption coefficients of phytoplankton in the red peak ($a_{ph}^{*}\left(676\right)$) (further details of this algorithm is beyond the scope of this paper but can be found in [6]). The coefficient $a_{ph}^{*}\left(676\right)$ is an essential input to the Step 2.

**Fig. 2:**
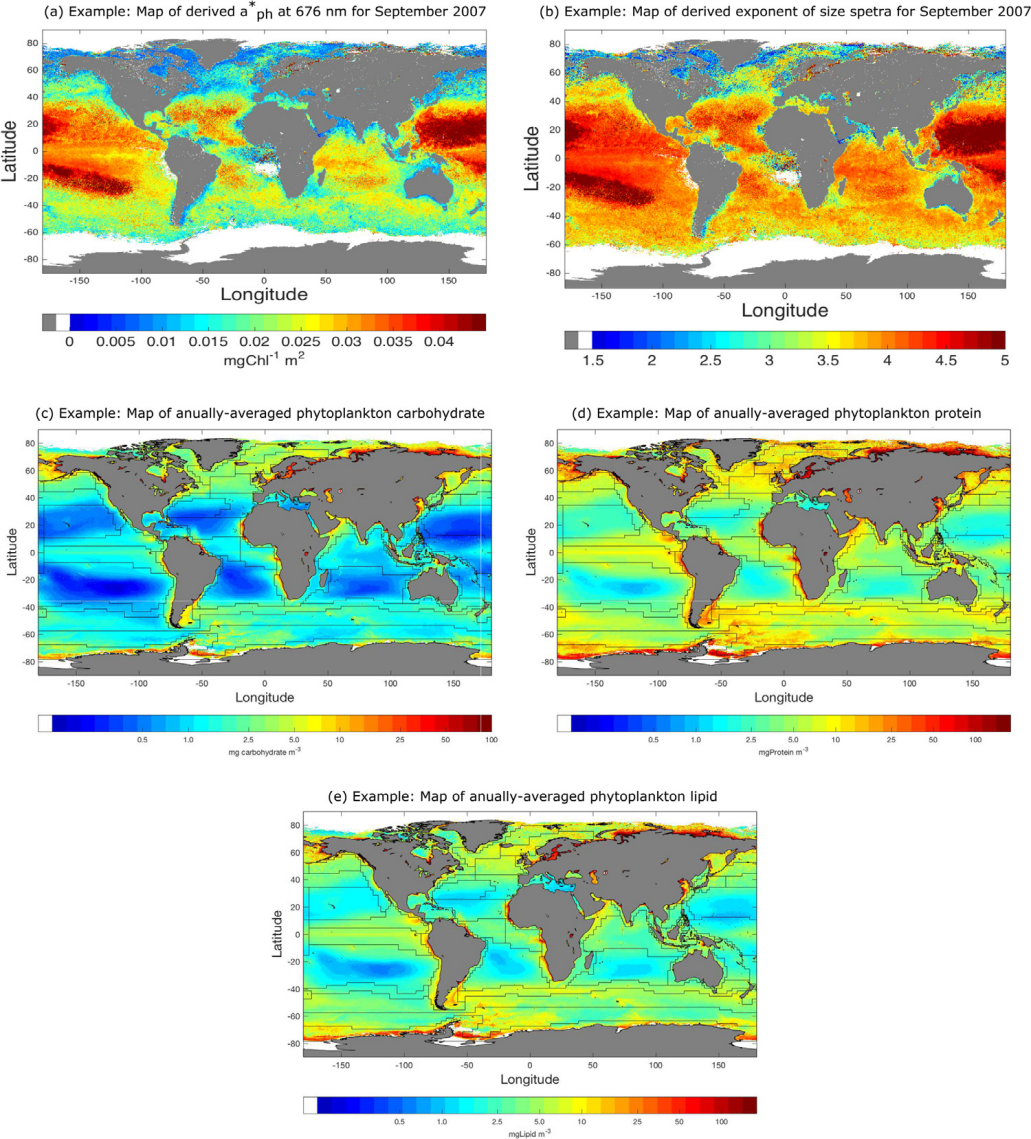
Examples of the method output. For illustration the method was applied to OC-CCI-v2 database (https://www.oceancolour.org). (a) Specific-absorption coefficient of phytoplankton at 676 nm i.e. $a_{ph}^{*}\left(676\right)$ was derived for September 2007 using IOP algorithms, which was then used to compute $a_{chl}^{*}\left(676\right)$ as described in Step 2. From this map, (b) the exponent of the phytoplankton size spectra (ξ) was computed as described in Step 3. Following Step 4, concentrations of phytoplankton (c) carbohydrate, (d) protein and (e) lipid were computed, and the annually-averaged values over 1997-2013 were shown in (c)–(e).

It should, however, be noted that alternative IOP algorithms can be used to retrieve phytoplankton absorption coefficients. The performance of the IOP algorithms are generally comparable to each other [6]. Alternative IOP algorithms that have been used include generalized ocean color inversion model for retrieving marine inherent optical properties (GIOP) [18], and ad-hoc empirical algorithms particularly for phytoplankton absorption coefficient at 676 nm. One constraint the users should note is that, often one may encounter ‘bad pixels’ (e.g. due to cloud or ice effect), at which semi-analytical inversion algorithms may not work. In such scenarios, a combination of semi-analytical and empirical inversion algorithms need to be used to generate $a_{ph}^{*}\left(676\right)$ maps over large area. For illustration, global maps of the derived $a_{ph}^{*}\left(676\right)$ values for a month is shown in Fig. 2 a.

### *Step 2: Method to compute of chlorophyll-specific absorption coefficient of phytoplankton from phytoplankton absorption spectra*

Chlorophyll-a is considered to be the principal light-absorbing pigment within phytoplankton cells, and so the absorption properties of phytoplankton are generally reported relative to the concentration of chlorophyll-a. The optical thickness $\rho_{c}$ (dimensionless) of a phytoplankton cell (assuming a homogeneous sphere with chlorophyll-a as the main absorbing pigment e.g. [8], [10]), for a ray of light of wavelength λ passing through the centre of cell, can be expressed as:

(1) $$\rho_{c}\left(\lambda \right)=D\times a_{ci}\left(\lambda \right)$$

where, D (say, in m) is the diameter the cell; $a_{ci}$ is a product of $c_{i}$ (in m${}^{-3}$ mgChl-a) - the intracellular chlorophyll-a concentration (i.e., concentration of chlorophyll-a per unit volume of the cell), and $a_{ci}^{*}\left(\lambda \right)$ (in m${}^{2}$ ${\left(\text{mg}\;\text{Chl-a}\right)}^{-1}$) - the specific absorption coefficient of chlorophyll-a inside the cell, such that, $a_{ci}^{*}\left(\lambda \right)=a_{ci}\left(\lambda \right)/c_{i}$ [10], [13]. The intracellular chlorophyll-a concentration ($c_{i}$) is derived from the isometric scaling between cell volume and chlorophyll-a per cell based on the equation $c_{i}=c_{0}\;D^{-m}\;$, and the magnitudes of the parameters m and $c_{0}$ are estimated as: $c_{0}=3.9\times 10^{6}$ (mg Chl-a m${}^{-2.94}$) and m=0.06 [13], so that $\rho_{c}\left(\lambda \right)=a_{ci}^{*}\left(\lambda \right)\;c_{0}\;D^{1-m}$.

Based on Duysens (1956) and van de Hulst (1957) the absorption efficiency $Q_{a}$ (dimensionless) of a cell, which is defined as the ratio of the light absorbed by the cell to the incident light, can be expressed as:

(2) $$Q_{a}\left(\rho_{c}\left(\lambda \right)\right)=1+\frac{2\;\exp\left(-\rho_{c}\left(\lambda \right)\right)}{\rho_{c}\left(\lambda \right)}+2\;\frac{\exp\left(-\rho_{c}\left(\lambda \right)\right)-1}{\rho_{c}{\left(\lambda \right)}^{2}}\;,$$

Now, the flattening effect $F(\rho_{c}\left(\lambda \right))$ (dimensionless), defined as the ratio of the absorption coefficient of a substance in discrete particles form suspended in the medium ($a_{p}\left(\lambda \right)$), to the absorption coefficient of the same substance dissolved uniformly in a medium ($a_{sol}\left(\lambda \right)$), can be expressed as:

(3) $$F\left(\rho_{c}\left(\lambda \right)\right)=\frac{a_{p}\left(\lambda \right)}{a_{sol}\left(\lambda \right)}=\frac{3\;Q_{a}\left(\rho_{c}\left(\lambda \right)\right)}{2\;\rho_{c}\left(\lambda \right)},$$

So, the specific absorption coefficient of chlorophyll-a $a_{chl}^{*}\left(\lambda \right)$ (in m${}^{2}$ ${\left(\text{mg}\;\text{Chl-a}\right)}^{-1}$) of intact phytoplankton cells in water can be expressed as:

(4) $$a_{chl}^{*}\left(\rho_{c}\left(\lambda \right)\right)=a_{ci}^{*}\left(\lambda \right)\times F\left(\rho_{c}\left(\lambda \right)\right)=\frac{3\;a_{ci}^{*}\left(\lambda \right)\;Q_{a}\left(\rho_{c}\left(\lambda \right)\right)}{\;2\rho_{c}\left(\lambda \right)}.$$

Although chlorophyll-a is considered as the main absorbing pigment inside a phytoplankton cell, auxiliary pigments may also contribute to the total phytoplankton absorption $a_{ph}\left(\lambda \right)$, especially at low chlorophyll concentrations. Decoupling the absorption by auxiliary pigments (say, $a_{u}\left(\lambda \right)$) and that by chlorophyll-a ($a_{chl}\left(\lambda \right)$), the total phytoplankton absorption $a_{ph}\left(\lambda \right)$ at any wavelength λ can be expressed as:

(5) $$a_{ph}\left(\lambda \right)=a_{chl}\left(\lambda \right)+a_{u}\left(\lambda \right).$$

The coefficients of phytoplankton absorption normalized to chlorophyll-a ($a_{ph}^{*}\left(\lambda \right)$), can then be obtained by dividing both sides of Eq. (5) by B, the concentration of chlorophyll-a:

(6) $$a_{ph}^{*}\left(\lambda \right)=\frac{a_{ph}\left(\lambda \right)}{B}=\frac{a_{chl}\left(\lambda \right)}{B}+\frac{a_{u}\left(\lambda \right)}{B}=a_{chl}^{*}\left(\lambda \right)+\frac{a_{u}\left(\lambda \right)}{B},$$

where, $a_{chl}^{*}\left(\lambda \right)$ is the specific absorption of chlorophyll-a, which is eventually a function of cell diameter as in Eq. (4). Usually the second term on the right-hand side of the Eq. (6) is ignored on the assumption that $a_{chl}^{*}\left(\lambda \right)\approx a_{ph}^{*}\left(\lambda \right)$, e.g., in the red absorption peak of chlorophyll-a (at λ≈676 nm). But Eq. (6) suggests that in low chlorophyll-a conditions, this assumption may overestimate the magnitude of $a_{chl}^{*}\left(\lambda \right)$ significantly. To overcome this uncertainty, Roy et al. [13] derived the following non-linear relationship between $a_{chl}^{*}\left(\lambda \right)$ and $a_{ph}^{*}\left(\lambda \right)$:

(7) $$a_{chl}^{*}\left(\lambda \right)=\frac{a_{ph}^{*}\left(\lambda \right)}{1+\sigma_{c}\left(\lambda \right)\;a_{ph}^{*}\left(\lambda \right)},$$

with magnitude of the parameter $\sigma_{c}\left(\lambda \right)$ derived as:

(8) $$\sigma_{c}\left(\lambda \right)=\left(\frac{1}{a_{ci}^{*}\left(\lambda \right)}-\frac{1}{a^{m}\left(\lambda \right)}\right).$$

Here, $a^{m}\left(\lambda \right)$ is the maximum value that $a_{ph}^{*}\left(\lambda \right)$ approaches to in a limiting condition, as the total absorption decreases. Using phytoplankton absorption spectra based on *in situ* measurements, Roy et al. [13] estimated the magnitude $a^{m}\left(\lambda \right)$ at λ=676 nm as $0.0412\;{\text{m}}^{2}{\left(\text{mgChl-a}\right)}^{-1}$. Additionally, for λ=676 nm, $a_{ci}^{*}\left(676\right)$ is taken as 0.028 ${\text{m}}^{2}\;{\left(\text{mg}\;\text{Chl-a}\right)}^{-1}$, based on the values reported for laboratory cultures. Eq. (7) suggests that at any given wavelength (say, 676 nm) chlorophyll-a specific absorption ($a_{chl}^{*}$) increases with phytoplankton absorption normalized to chlorophyll-a ($a_{ph}^{*}$) to a saturation level, and the parameter $\sigma_{c}\left(\lambda \right)$ determines how slowly the saturation level is reached.

### *Step 3: Method to compute phytoplankton size spectra from chlorophyll-specific absorption coefficient*

In seawater, phytoplankton cells are assumed to follow a particle-size distribution defined by a Jung-type power law [7], [14]. Under this assumption, the differential number concentration N of phytoplankton cells per unit volume of seawater with a cell diameter of D is given by:

(9) $$N\left(D\right)=kD^{-\xi },$$

where ξ represents the exponent of the phytoplankton size spectrum and k is the constant of power law related to the abundance of the phytoplankton population. So, within the diameter range $\left[D_{min},\;D_{max}\right]$, the concentration of phytoplankton chlorophyll-a (B) can be integrated as a product of the volume of the cell ($\frac{\pi }{6}\;D^{3}$), the intracellular chlorophyll-a concentration ($c_{i}$), and the total number of cells $N_{T}$ per unit volume of seawater within that diameter range, as follows:

(10) $$B_{total}=\int_{D_{min}}^{D_{max}}\;\left[\left(\frac{\pi }{6}\;D^{3}\right)\;\left(c_{0}\;D^{-m}\right)\;\left(kD^{-\xi }\right)\right]\;dD=\left(\frac{\pi }{6}\;k\;c_{0}\right)\frac{D_{max}^{4-\xi -m}-D_{min}^{4-\xi -m}}{4-\xi -m}.$$

Similarly, at a wavelength λ=676 nm, the total absorption by chlorophyll-a due to the phytoplankton cells in the diameter range $\left[D_{min},\;D_{max}\right]$ can be obtained from Eq. (4) by integration, as follows:

(11) $$a_{chl}\left(676\right)=\int_{D_{min}}^{D_{max}}\;\left[\left(\frac{\pi }{6}\;D^{3}\right)\;\left(c_{0}\;D^{-m}\right)\;\left(kD^{-\xi }\right)\;a_{chl}^{*}\left(\rho_{c}\left(676\right)\right)\right]\;dD=\left(\frac{\pi }{6}\;k\;c_{0}\right)\;\int_{D_{min}}^{D_{max}}\;\left[D^{3-\xi -m}\;a_{chl}^{*}\left(\rho_{c}\left(676\right)\right)\right]\;dD.$$

Therefore, the specific absorption of chlorophyll-a at λ=676 nm, due to phytoplankton cells in the diameter range $\left[D_{min},\;D_{max}\right]$, can be express as:

(12) $$a_{chl}^{*}\left(676\right)=\frac{a_{chl}\left(676\right)}{B_{total}}=\frac{4-\xi -m}{D_{max}^{4-\xi -m}-D_{min}^{4-\xi -m}}\;\int_{D_{min}}^{D_{max}}\;\left[D^{3-\xi -m}\;a_{chl}^{*}\left(\rho_{c}\left(676\right)\right)\right]\;dD,$$

with the limiting condition:

$a_{chl}^{*}\left(676\right)\to \left(\frac{1}{\log_{e}\left(D_{max}/D_{min}\right)}\;\int_{D_{min}}^{D_{max}}\;\left[D^{3-\xi -m}\;a_{chl}^{*}\left(\rho_{c}\left(676\right)\right)\right]\;dD\right)$, as ξ→(4−m).

The coefficients of phytoplankton absorption normalized to chlorophyll-a at 676 nm ($a_{ph}^{*}\left(676\right)$) is obtained from satellite-derived chlorophyll and $a_{ph}\left(676\right)$. Eq. (7) is then used to convert $a_{ph}^{*}\left(676\right)$ into $a_{chl}^{*}\left(676\right)$ values, which are then used on the left-hand side of Eq. (12), to numerically estimate ξ over a given range of diameter $\left[D_{min},\;\;D_{max}\right]$. The magnitude of ξ is estimated iteratively within an optimisation method, where the integral in the right-hand side of Eq. (12) is calculated numerically for an initial value of ξ, which is then optimized to minimize the differences between the two sides of Eq. (12). Corresponding to a given value of $a_{ph}^{*}\left(\lambda \right)$, the estimate of ξ can be obtained through an optimisation method chosen by the user. Given the deterministic relationship, the estimates of ξ would be unique and independent of the choice of the optimisation method. Therefore, the user is left to choose the optimisation method and coding platform/package. For example, Roy et al. [12] used a single-variable bounded nonlinear-function minimization method based on golden section search and parabolic interpolation implemented on MATLAB(R) [3]. For illustration, a global map of the derived ξ values for a month is shown in Fig. 2 b.

### *Step 4: Method to compute of phytoplankton macromolecular concentration from phytoplankton size spectra*

The allometric relationship between the cellular concentration of a phytoplankton macromolecule (e.g. carbohydrate, protein or lipid, with the notation ${\left[M\right]}_{cell}$ in the units of pg cell${}^{-1}$) and the volume of a phytoplankton cell ($V_{cell}$, in μm${}^{3}$) is given by: ${\left[M\right]}_{cell}=a_{M}\;V_{cell}^{b_{M}}$; where $a_{M}$, $b_{M}$ are the allometric parameters with magnitudes specific to the macromolecule M. The allometric parameters $a_{M}$ and $b_{M}$ are fixed based on the literature e.g. [2] . The total concentration of the macromolecule M (in mg m${}^{-3}$) due to all phytoplankton cells within a diameter range $\left[D_{min},\;D_{max}\right]$ can be integrated as a product of the number of cells N(D) within that diameter range and the cellular concentration ${\left[M\right]}_{cell}$ [11]:

(13) $${\left[M\right]}_{total}=\int_{D_{min}}^{D_{max}}\;\left[N\left(D\right)\;{\left[M\right]}_{cell}\right]\;dD=10^{-9}\;k\;a_{M}\;{\left(10^{18}\;\frac{\pi }{6}\right)}^{b_{M}}\;\left(\frac{D_{max}^{3b_{M}-\xi +1}-D_{min}^{3b_{M}-\xi +1}}{3b_{M}-\xi +1}\right);$$

with the limiting condition: ${\left[M\right]}_{total}\;\to \;\left[10^{-9}\;k\;a_{M}\;{\left(10^{18}\;\pi /6\right)}^{b_{M}}\;\log_{e}\left(D_{max}/D_{min}\right)\right]$, as $\xi \;\to \;(3b_{M}+1)$, applied to avoid division by zero. The factors $10^{-9}$ and $10^{18}$ are associated with the conversions of units from pg to mg, and m${}^{3}$ to μm${}^{3}$, respectively. Now, combining Eqs. (10) and (13), the ratio of the macromolecular concentration to the concentration chlorophyll ($\chi_{M}$) can be expressed as:

(14) $$\chi_{M}=\frac{{\left[M\right]}_{total}}{B_{total}}=\frac{10^{-9}\;a_{M}\;{\left(10^{18}\;\pi /6\right)}^{b_{M}}}{\left(\pi /6\right)\;c_{0}}\;\left(\frac{D_{max}^{3b_{M}-\xi +1}-D_{min}^{3b_{M}-\xi +1}}{D_{max}^{4-\xi -m}-D_{min}^{4-\xi -m}}\right)\;\left(\frac{4-\xi -m}{3b_{M}-\xi +1}\right).$$

By computing the ratio, the parameter k is removed from the expression of macromolecule-to-chlorophyll ratio $\chi_{M}$ in Eq. (14). This way $\chi_{M}$ is first computed from Eq. (14) with inputs of ξ from Step 3, and then $M_{total}$ is computed from the observed value of $B_{total}$ as follows:

(15) $$M_{total}=\chi_{M}\;B_{total}.$$

For illustration, global maps of the derived annually-averaged concentrations of phytoplankton carbohydrate, protein and lipid are shown in Fig. 2 c–e. The total concentration of the macromolecules can further be partitioned into macromolecules in n non-overlapping phytoplankton size classes (PSC) defined by cell-diameter ranges $\left[D_{i},\;D_{j}\right]$ with 0≤i<j≤n, so that ${\left[M\right]}_{total}=\sum \;{\left[M\right]}_{ij}$, where ${\left[M\right]}_{ij}$ is the macromolecular concentration within the size class [i,j]. Using Eq. (15), ${\left[M\right]}_{ij}$ can be expressed as:

(16) $${\left[M\right]}_{ij}=\chi_{M\;ij}\;B_{ij},$$

with $\chi_{M\;ij}$ and $B_{ij}$, respectively, are the macromolecule-to-chlorophyll ratio and the concentration of chlorophyll $B_{ij}$ in the size class $\left[D_{i},\;D_{j}\right]$, where $\chi_{M\;ij}$ follows from Eq. (14):

(17) $$\chi_{M\;ij}=\frac{10^{-9}\;a_{M}\;{\left(10^{18}\;\pi /6\right)}^{b_{M}}}{\left(\pi /6\right)\;c_{0}}\;\left[\frac{D_{j}^{3b_{M}-\xi +1}-D_{i}^{3b_{M}-\xi +1}}{D_{j}^{4-\xi -m}-D_{i}^{4-\xi -m}}\right]\;\left[\frac{4-\xi -m}{3b_{M}-\xi +1}\right],$$

and based on Roy et al. [12], $B_{ij}/B_{total}$ is given by: $\left(\frac{D_{j}^{4-\xi -m}-D_{i}^{4-\xi -m}}{D_{max}^{4-\xi -m}-D_{min}^{4-\xi -m}}\right)$, so that,

(18) $${\left[M\right]}_{ij}=\chi_{M\;ij}\;B_{ij}=\chi_{M\;ij}\;\left(\frac{D_{j}^{4-\xi -m}-D_{i}^{4-\xi -m}}{D_{max}^{4-\xi -m}-D_{min}^{4-\xi -m}}\right)\;B_{total};$$

Therefore, ${\left[M\right]}_{total}$ can also be expressed as:

(19) $${\left[M\right]}_{total}=\sum_{i=0,\;j=i+1}^{i=n-1,\;j=n}{\left[M\right]}_{ij}=\frac{B_{total}}{D_{max}^{4-\xi -m}-D_{min}^{4-\xi -m}}\;\sum_{i=0,\;j=i+1}^{i=n-1,\;j=n}\left[\chi_{M\;ij}\;\left(D_{j}^{4-\xi -m}-D_{i}^{4-\xi -m}\right)\right],$$

and the fraction of ${\left[M\right]}_{ij}$ to ${\left[M\right]}_{total}$ can be computed as:

(20) $$F_{M,ij}=\frac{{\left[M\right]}_{ij}}{{\left[M\right]}_{total}}=\frac{\chi_{M\;ij}\left(D_{j}^{4-\xi -m}-D_{i}^{4-\xi -m}\right)}{\sum_{i=0,\;j=i+1}^{i=n-1,\;j=n}\left[\chi_{M\;ij}\;\left(D_{j}^{4-\xi -m}-D_{i}^{4-\xi -m}\right)\right]}.$$

Any number of PSCs can be implemented within the equations above. In particular, to obtain macromolecular concentrations in three major PSCs, i.e., picoplankton, nanoplankton and microplankton, the diameter bounds need to be specified as: $D_{0}=0.25$ μm, $D_{1}=2$ μm, $D_{2}=20$ μm, and $D_{3}=50$ μm (e.g. [12], [15], [17]). For illustration, a MATLAB script to compute macromolecular concentrations in total and those partitioned into different size classes can be found in the supplementary materials.

### *Computation of uncertainty*

The estimates of macromolecular concentrations based on the method described above ideally should be validated with direct *in situ* measurements. In the absence of primary *in situ* measurements (which is often the case), uncertainty in the estimates can be computed as a function of the satellite inputs to the algorithm. These uncertainty levels are valuable for the users, given that the most prominent sources of uncertainties are associated with the satellite products used as inputs to the model e.g. chlorophyll-a and phytoplankton absorption coefficients. To quantify the overall uncertainty levels in the satellite-derived estimates of macromolecular concentrations, a theoretical sensitivity analysis with respect to most important input parameters can be carried out. The allometric parameters ($a_{M}$ and $b_{M}$) and the derived values of ξ, determine the estimates of the macromolecular concentrations as in Step 4. The estimates ξ further relies on $a_{ph}^{*}$. The relative sensitivity $M_{total}$, expressed as $\frac{\Delta M_{total}}{M_{total}}$ is then a combined function of $\frac{\Delta \;\xi }{\xi }$, $\frac{\Delta \;a_{M}}{a_{M}}$, and $\frac{\Delta \;b_{M}}{b_{M}}$, and can be computed using Eqs. ((10)–(14)) as follows:

(21) $$\begin{array}{ccc} \frac{\Delta M_{total}}{M_{total}} & = & \frac{\Delta \;a_{M}}{a_{M}}+\left(\log\left(\left(10^{18}\right)\;\pi /6\right)\right)\;b_{M}\;\frac{\Delta \;b_{M}}{b_{M}}\\ & & +(\left(D_{max}^{3\;b_{M}-\xi +1}\right)\;\log\left(D_{max}\right)\\ & & -\left(D_{min}^{3\;b_{M}-\xi +1}\right)\;\log\left(D_{min}\right))\;\left(3\;b_{M}\;\frac{\Delta \;b_{M}}{b_{M}}-\xi \;\frac{\Delta \;\xi }{\xi }\right)/\left(D_{max}^{3\;b_{M}-\xi +1}-\left(D_{min}^{3\;b_{M}-\xi +1}\right)\right)\\ & & -\left(\left(\left(D_{max}^{4-\xi -m}\right)\;\log\left(D_{max}\right)\right)-\left(\left(D_{min}^{4-\xi -m}\right)\;\log\left(D_{min}\right)\right)\right)\;\left(-\xi \;\frac{\Delta \;\xi }{\xi }\right)./\left(\left(D_{max}^{4-\xi -m}\right)-\left(D_{min}^{4-\xi -m}\right)\right)\\ & & -\frac{1}{4-\xi -m}\;\xi \;\frac{\Delta \;\xi }{\xi }-\frac{1}{3\;b_{M}-\xi +1}\;\left(3\;b_{M}*\frac{\Delta \;b_{M}}{b_{M}}-\xi \;\frac{\Delta \;\xi }{\xi }\right). \end{array}$$

**Fig. 3:**
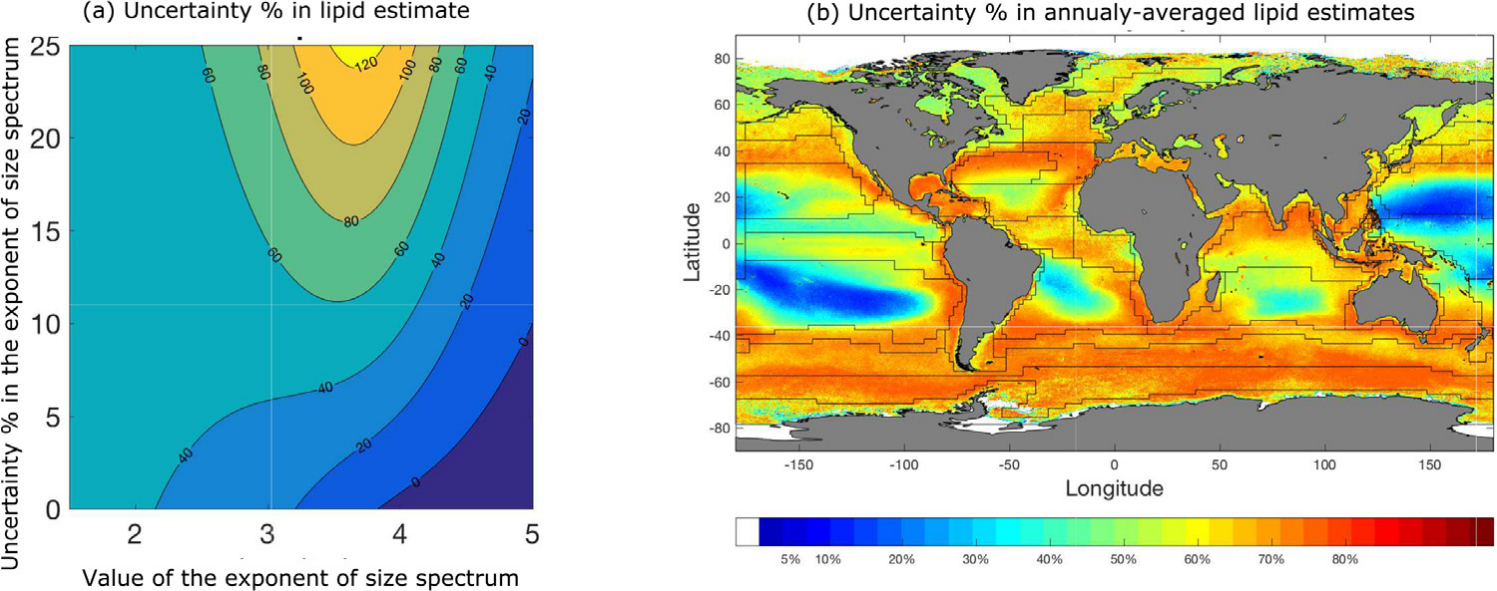
Example of uncertainty level in estimated macromolecular concentrations based on the method described.

For illustration, a MATLAB script to compute the uncertainties based on the above equation can be found in the supplementary materials. Generally on a global scale, $\frac{\Delta \;\xi }{\xi }$ can be considered within the range 0–25% [12], and for $\frac{\Delta \;a_{M}}{a_{M}}$ and $\frac{\Delta \;b_{M}}{b_{M}}$, the half of the 95% spread with respect to their mean levels reported in [2] can be considered. The computed overall uncertainty $\frac{\Delta M_{total}}{M_{total}}$ can be mapped pixel-by-pixel over the oceanographic regions. To illustrate the uncertainty computations, Fig.  3 shows the computed uncertainty in the estimates of phytoplankton lipid concentration, and maps those for annually-averaged estimates of phytoplankton lipid.

## Declaration of Competing Interest

The author declares no conflicts of interest.

## Appendix A Supplementary materials

Supplementary Data S1 Supplementary Raw Research Data. This is open data under the CC BY license http://creativecommons.org/licenses/by/4.0/ Supplementary Data S1
