# Supplementary material for: Algorithmic procedure for retrieving calorific contents of marine phytoplankton from space
Source: MethodsX. 2021 Nov 16;8:101579. doi: 10.1016/j.mex.2021.101579 (PMC8720915; doi:10.1016/j.mex.2021.101579)
Supplement: Supplementary Data S1 — Supplementary Raw Research Data. This is open data under the CC BY license http://creativecommons.org/licenses/by/4.0/ [file mmc1.docx]

Supplementary material:

MATLAB script of the method

**Algorithmic procedure for retrieving calorific contents of marine phytoplankton from space**

**Shovonlal Roy**

Department of Geography and Environmental Science, University of Reading,

Whiteknights, Reading RG6 6AB, U.K.

Email: [shovonlal.roy@reading.ac.uk](mailto:shovonlal.roy@reading.ac.uk)

This is a script to implement the method using MATLAB. To build an executable script the users are advised to either code the steps onto the coding platform of their choice following the method described, or adapt this part of the partial MATLAB script to the satellite data and Inherent Optical Properties algorithms of their choice.

*% This script outlines the calculations of phytoplankton carbohydrate, protein and lipid concentration based on the methods described in the paper: “Algorithmic procedure for retrieving calorific contents of marine phytoplankton from space”.*

*%The script is based on MATLAB. It includes the main functions %“phytoplankton_carbo_prot_lip_dmin_dmax” that calculates the %concentrations of carbohydrate, protein and lipid; and the function*

*%uncertainty_carbo_prot_lipid” that computes the uncertainty in the %estimates.*

*%phy_CPL = concentrations of phytoplankton carbohydrate, protein, lipid*

*%Dmin= lowest value in the cell diameter range considered*

*%Dmax= highest value in the cell diameter range considered*

*%chl_data = Chlorophyll concentrations from the satellite sensor of choice*

*%xi_data0=Exponents of phytoplankton size spectrum*

*%phy_carbo = Concentration of total phytoplankton carbohydrate*

*%phy_prot = Concentration of total phytoplankton protein*

*%phy_lip = Concentration of total phytoplankton lipid*

*%pico_carbo = Concentration of phytoplankton carbohydrate pico-group*

*%pico_prot= Concentration of phytoplankton protein pico-group*

*%pico_lip = Concentration of phytoplankton lipid pico-group*

*%nano_carbo = Concentration of phytoplankton carbohydrate nano-group*

*%nano_prot = Concentration of phytoplankton protein nano-group*

*%nano_lip = Concentration of phytoplankton lipid nano-group*

*%micro_carbo = Concentration of phytoplankton carbohydrate micro-group*

*%micro_prot= Concentration of phytoplankton protein micro-group*

*%micro_lip = Concentration of phytoplankton lipid micro-group*

function [phy_carbo, phy_prot, phy_lip, pico_carbo, pico_prot, pico_lip, nano_carbo, nano_prot, nano_lip, micro_carbo, micro_prot, micro_lip] = phytoplankton_carbo_prot_lip_dmin_dmax(chl_data,xi_data0,dmin,dmax)

*%parameters*

Dmin=0.25*10^(-6);

Dmax=50*10^(-6);

c0 = 3.9*10^6; m=0.06;

carbo_prot_lip_all_a =[0.72 0.70 0.73; 1.09 1.07 1.10; 0.80 0.79 0.81];

carbo_prot_lip_all_b =[0.93 0.87 0.99; 0.83 0.80 0.86; 0.80 0.76 0.85];

phy_carbo= zeros(length(xi_data0),1);

phy_prot= zeros(length(xi_data0),1);

phy_lip = zeros(length(xi_data0),1);

pico_carbo = zeros(length(xi_data0),1);

pico_prot = zeros(length(xi_data0),1);

pico_lip = zeros(length(xi_data0),1);

nano_carbo = zeros(length(xi_data0),1);

nano_prot = zeros(length(xi_data0),1);

nano_lip = zeros(length(xi_data0),1);

micro_carbo = zeros(length(xi_data0),1);

micro_prot = zeros(length(xi_data0),1);

micro_lip = zeros(length(xi_data0),1)

b1=carbo_prot_lip_all_b(1, 1);

a10=carbo_prot_lip_all_a(1,1);

a1= 10.^(a10-2*b1);

b2=carbo_prot_lip_all_b(1, 1);

a20=carbo_prot_lip_all_a(1,1);

a2= 10.^(a20-2*b2);

b3=carbo_prot_lip_all_b(1, 1);

a30=carbo_prot_lip_all_a(1,1);

a3= 10.^(a30-2*b3);

*%%%xi_data0 - for non-zero denominator*

xi_data=round(xi_data0*10^4)/(10^4);

xi_data(xi_data0==round((3*b1+1)*10^4)/(10^4))=(3*b1+1+0.001);

xi_data(xi_data0==3.94)=3.94005;

*%In the following part ‘mm’ should be replaced by ‘carbo’, ‘prot’ and ‘lip’, as required, and the %corresponding ‘a’ and ‘b’ values should be used*

[mm_chl_pic,mm_frac_pic0]= mm_chl_mm_frac0(xi_data,c0,m,a1,b1,Dmin,Dmax, dmin, 2*10^(-6));

[mm_chl_nano, mm_frac_nano0]= mm_chl_mm_frac0(xi_data,c0,m,a1,b1,Dmin,Dmax, 2*10^(-6), 20*10^(-6));

[mm_chl_mic, mm_frac_mic0]=mm_chl_mm_frac0(xi_data,c0,m,a1,b1,Dmin,Dmax, 20*10^(-6), Dmax);

total_mm_frac = mm_frac_pic0 + mm_frac_nano0 + mm_frac_mic0;

mm_frac_pic= mm_frac_pic0./total_mm_frac;

mm_frac_nano= mm_frac_nano0./total_mm_frac;

mm_frac_mic= mm_frac_mic0./total_mm_frac;

%%%cchl of pico

cc_pico_mm = mm_chl_pic.*chl_data.*mm_frac_pic;

cc_nano_mm = mm_chl_nano.*chl_data.*mm_frac_nano;

cc_micro_mm = mm_chl_mic.*chl_data.*mm_frac_mic;

phy_mm = cc_pico+cc_nano+cc_micro;

%%%

phy_carbo(:)= phy_carbo;

pico_carbo(:)=cc_pico_carbo;

cc_nano_carbo(:)=cc_nano_carbo;

cc_micro_carbo(:)=cc_micro_carbo;

phy_prot (:)= phy_prot;

pico_prot (:)=cc_pico_prot;

cc_nano_prot (:)=cc_nano_prot;

cc_micro_prot(:)=cc_micro_prot;

phy_lip(:)= phy_lip;

pico_lip(:)=cc_pico_lip;

cc_nano_lip(:)=cc_nano_lip;

cc_micro_lip(:)=cc_micro_lip;

function [mm_chl, mm_frac]=mm_chl_mm_frac0(xi,c0,m,a,b,Dmin,Dmax, Di, Dj)

mm_chl = ((10^(-9))*a.*(((10^18)*(pi/6)).^b)./(pi*c0/6)).* (((Dj.^(3*b-xi+1))-(Di.^(3*b-xi+1)))./((Dj.^(4-xi-m))-(Di.^(4-xi-m)))).*((4-xi-m)./(3*b-xi+1));

mm_frac = mm_chl.*(((Dj.^(4-xi-m))-(Di.^(4-xi-m)))./((Dmax.^(4-xi-m))-(Dmin.^(4-xi-m))));

*%This part of the program computes uncertainty in estimates of macromolecules (carbo/prot/lipid) %due 'a', 'b' and 'xi'*

function perc_error_mm=uncertainty_carbo_prot_lipid(allo_a, allo_b, er_a, er_b, xi_data, er_xi)

Dmin=0.2*10^(-6);

Dmax=50*10^(-6);

m=0.06;

perc_error_b =er_b;

perc_error_a =er_a;

perc_error_xi= er_xi;

xi_data=round(xi_data0*10^4)/(10^4);

xi_data(xi_data==round((3*allo_b+1)*10^4)/(10^4))=(3*allo_b+1+0.001);

xi_data(xi_data==3.94)=3.9405;

perc_error_carbon = perc_error_a + (log((10^18)*pi/6)).*allo_b.*perc_error_b...

+ ((Dmax.^(3*allo_b-xi_data+1)).*log(Dmax) - (Dmin.^(3*allo_b-xi_data+1)).*log(Dmin)).*(3*allo_b.*perc_error_b - xi_data.*perc_error_xi)./(Dmax.^(3*allo_b-xi_data+1)-(Dmin.^(3*allo_b-xi_data+1))) - (((Dmax.^(4-xi_data-m)).*log(Dmax))-((Dmin.^(4-xi_data-m)).*log(Dmin))).*(-xi_data.*perc_error_xi)./((Dmax.^(4-xi_data-m))- (Dmin.^(4-xi_data-m))) + (-1./(4-xi_data-m)).*xi_data.*perc_error_xi - (1./(3.*allo_b-xi_data+1)).*(3*allo_b.*perc_error_b -xi_data.*perc_error_xi);
